# Progress in Diseases of the Blood

**Published:** 1902-12-13

**Authors:** 


### Progress in Diseases of the Blood.

(*Concluded from page* 163.)

**Hodgkin's Disease.**—Wende[7] gives an instance of the change of this disease into leukæmia. A male, aged 26, was affected with glandular swellings and also with nodules and greyish-yellow patches of pigment in the skin and small areas of induration. The blood was at first normal. Later on he began to fail, the blood became anæmic with 96·2 per cent. of lymphocytes, and some normoblasts. Hæmorrhages and petechiæ became numerous and death occurred in seven months. The cutaneous lesions were formed of lymphoid tissue, and the glands generally were crowded with similar cells. The spleen weighed 46 ounces and, like the liver and kidneys, showed lymphoid infiltration. Typical lymphatic leukæmia does occasionally, as in this case, cause affections of the skin. T. Law-Webb[8] mentions that while two forms of Hodgkin's disease exist, one (*a*) showing only some enlargement of the lymphatic glands, and the other (*b*) a development of adenoid tissue where little or none normally exists, as well as splenic hypertrophy, anæmia, and a blood-state like lymphatic leukæmia; still the mild simple form generally passes into the severe one at last. The blood changes, however, are uncertain, and may be insignificant. If the leukæmic type is present a difference from the latter disease exists in the smallness of the lymphocytes, the scantiness of their protoplasm and the rarity of mitotic changes in them, whereas in this leukæmia one lymphocyte in five or six has a dividing nucleus. Indeed, he thinks that the lymphocytosis may be only accidental in Hodgkin's disease and due to over-compensation by the new adenoid tissue which arises after the destruction of the glands. When the spleen has been removed a similar growth of new adenoid tissue and lymphocytosis often takes place as a normal process of repair.

**Banti's Disease.**—In addition to the references given above, under the heading of splenic anæmia, interesting papers have appeared by W. Murrell and J. Barr. Barr[9] reports three cases—the first patient, a male aged 48, dated his illness from an accident four years previously, and looked like a man suffering from cirrhosis of the liver. The spleen was enormously enlarged, and the liver increased. There was ascites œdema and fluid in one pleura. The blood showed at first that the hæmoglobin was 40 per cent. and red cells 2,400,000, with an excess of lymphocytes. The second patient, a male aged 42, had suffered from hæmatemesis twice during five years. His skin was yellow and the liver and spleen enlarged. The gums were spongy and bled easily. The red cells were normal, but the hæmoglobin reduced. He suffered later on from erythro-melalgia of the feet, and pain and swelling in the knees and ankles. There was no ascites present. The third, a male aged 38, gave a history of an injury five years before. Splenic enlargement, emaciation, slight jaundice, melæna, great prostration, ascites and fluid in the pleura, were noted. Considerable improvement took place in 11 months, and the blood then showed red cells normal, though they had previously numbered 2,800,000, and hæmoglobin 85 per cent., with lymphocytes 18 and eosinopheles 7 per cent. All the patients were alive in August, 1902. Barr considers that a vaso-motor paresis of the splanchnic area is an important factor in the disease, and notices the very low blood pressure in all these patients. This does not seem to occur in simple cirrhosis of the liver. These patients improved under treatment, and as the spleens became smaller the red cells increased to normal and even above it. The hæmoglobin was reduced in all. Treatment comprised intestinal antisepsis, and various means for improving the nutrition and raising the blood pressure generally. Such drugs as digitalis strychnia and perchloride of iron with calcium chloride were employed. W. Murrell[10] mentions that Banti did not speak of cirrhosis of the liver and spleen, but tumefaction and hypertrophy, the spleen being first affected, Murrell regards the disease as synonymous with splenic anæmia, and speaks of the fewness both of the red and white cells, the hæmorrhages, pyrexia, the changes in the spleen and sometimes in the liver, and the bad prognosis. He favours the establishment of a collateral circulation in place of splenectomy, if surgical interference is decided on. He finds great value in ascitic cases from 15 grain doses of Turpeth (Indian Addendum B.P., 1900) in place of jalap powder as a purgative, and believes that counter-irritants, such as capsicum ointment, aid in the enlargement of the superficial abdominal veins.

**The Œdema of Anæmia.**—J. Houston[11] has made an interesting investigation on this subject. While the œdema of Bright's disease comes and goes without simultaneous changes in the hæmoglobin, the œdema of anæmia seems to depend on the relative excess of water in the blood. The absence of loss of weight in anæmic conditions depends on abnormal accumulation of fluid in the blood and tissues. The first stage of cure, especially in chlorosis, consists in getting rid of this excess. The occurrence of hæmorrhages, diarrhœa, etc., in severe anæmias seems to be an effort to get rid of the excess of fluid. Frequent comparisons of the weight of the patient and of the hæmoglobin value give interesting results. A gain of weight in pernicious anæmia without a gain of hæmoglobin is unfavourable, but sometimes marks a critical stage followed by concentration of the blood.

[7] Lancet. March 15. [8] Brit. Med. Jour., Sept. 27. [9] Lancet, Aug. 23. [10] Clin. Jour., Aug. 6. [11] Brit. Med. Jour., June 14.

